# Ultrastructural Changes in the Midgut of Brazilian Native Stingless Bee *Melipona scutellaris* Exposed to Fungicide Pyraclostrobin

**DOI:** 10.3390/toxics11121028

**Published:** 2023-12-18

**Authors:** Caio E. C. Domingues, Lais V. B. Inoue, Aleš Gregorc, Leticia S. Ansaloni, Osmar Malaspina, Elaine C. Mathias da Silva

**Affiliations:** 1 Faculty of Agriculture and Life Sciences, University of Maribor, Pivola 10, 2311 Hoče, Slovenialeticia.salvioni1@um.si (L.S.A.); 2 Centro de Estudos de Insetos Sociais (CEIS), Departamento de Biologia Geral e Aplicada, Instituto de Biociências (IB), Universidade Estadual Paulista (UNESP)—“Júlio de Mesquita Filho”, Rio Claro 13506-900, SP, Brazil; lais.inoue@unesp.br (L.V.B.I.); osmar.malaspina@unesp.br (O.M.); 3 Laboratório de Ecotoxicologia e Análise de Integridade Ambiental (LEIA), Departamento de Biologia (DBio), Universidade Federal de São Carlos (UFSCar), Sorocaba 18052-780, SP, Brazil; elaine@ufscar.br

**Keywords:** digestive tract, Meliponini, mitochondria, morphology, strobilurin, sublethal effects

## Abstract

*Melipona scutellaris* is a Brazilian stingless bee that is important for pollinating wild flora and agriculture crops. Fungicides have been widely used in agriculture, and floral residues can affect forager bees. The goal of our study was to evaluate the effects of sublethal concentrations of pyraclostrobin on the midgut ultrastructure of *M. scutellaris* forager workers. The bees were collected from three non-parental colonies and kept under laboratory conditions. The bees were orally exposed continuously for five days to pyraclostrobin in syrup at concentrations of 0.125 ng a.i./µL (FG1) and 0.005 ng a.i./µL (FG2). The control bees (CTL) were fed a no-fungicide sucrose solution, and the acetone solvent control bees (CAC) received a sucrose solution containing acetone. At the end of the exposure, the midguts were sampled, fixed in Karnovsky solution, and routinely processed for transmission electron microscopy. Ultrastructural analysis demonstrated that both the fungicide concentrations altered the midgut, such as cytoplasmic vacuolization (more intense in FG1), the presence of an atypical nuclear morphology, and slightly dilated mitochondrial cristae in the bees from the FG1 and FG2 groups (both more intense in FG1). Additionally, there was an alteration in the ultrastructure of the spherocrystals (FG1), which could be the result of cellular metabolism impairment and the excretion of toxic metabolites in the digestive cells as a response to fungicide exposure. The results indicate that ingested pyraclostrobin induced cytotoxic effects in the midgut of native stingless bees. These cellular ultrastructural responses of the midgut are a prelude to a reduced survival rate, as observed in previous studies.

## 1. Introduction

Stingless bees are a large and diverse bee group belonging to the Meliponini tribe, of which around 550 species and 58 genera have been described worldwide [1,2]. As well as honey bees (*Apis mellifera*, Linnaeus, 1758), stingless bees are a member of the Apidae family and are the largest group of eusocial bees [3]. The geographical distribution of these bees is predominantly in tropical and subtropical regions, and they can be found in Africa [4], America [5], the Indo-Malayan region, and Australasia [2]. The distribution of stingless bees in these regions is closely related to the diversity of available flora (preferred plant families) [6].

In Brazil, there is a high diversity of about 250 described stingless bee species belonging to 29 genera, of which 20% are endemic [5]. Stingless bees are known for some remarkable characteristics, such as the incapacity to sting with a vestigial sting, well-developed defense strategies, morphological diversity, a variety of nest architectures, and importance for humans and the environment [2,7].

These native bee species are essential to conserving native flora [6,8,9,10] and are important for several crops [11,12,13,14]. Nowadays, the breeding and care of stingless bees (Meliponiculture) have become popular [15], which consequently has increased the commercialization and research of stingless bee products like honey, pollen, and propolis [16]. Additionally, many bioactive compounds, such as honey, propolis and geopropolis, with therapeutic properties that can contribute to treating human diseases, such as anti-inflammatory, antioxidant, and antimicrobial effects, have been discovered [17,18,19,20].

In the same way as previously mentioned, *Melipona scutellaris* (Latreille, 1811; common name—Northeast Uruçu) is an important bee species native to northeast Brazil [21], mainly due to pollination services [8] and honey production since its singular aroma and flavor are highly appreciated. The antibacterial properties of honey [22], and the antiproliferative constituents of geopropolis [23], have been described. Although *M. scutellaris* is an essential and relevant species, it is listed in the *Brazil Red Book of Threatened Species of Fauna* [24]. According to Toledo-Hernández et al. [25], many factors threaten stingless bees, highlighting pesticides (insecticides, herbicides, fungicides, biopesticides, and fertilizers), transgenic crops, deforestation, diseases and pests, competition for food resources, and climate change. This is worrying since stingless bees are not as well studied as honey bees [26].

The increasing use of fungicides, worldwide, for many years [27], means bees are more exposed to fungicides by aerial spraying or ingesting floral resources containing their residues [28]. Raimets et al. [29] detected several fungicides in beekeeping matrices. In that regard, pyraclostrobin is one of the most relevant strobilurin fungicides used in some crops that stingless bees visit (coffee, eucalyptus, and pepper) and has also been detected in pollen [30], nectar [31], and beebread [32]. Due to the inhibition of mitochondrial respiration [33], the fungicide pyraclostrobin can affect essential functions of bee physiology. A few studies have shown the side effects of fungicides on non-target stingless bees [34,35,36,37,38], but there is still a considerable gap between insecticide and fungicide studies [25].

Additionally, there is a lack of knowledge about the sublethal effects of fungicides on the digestive tracts of bees at a cellular level, which is responsible for vital nutritional functions [39,40] and is an entrance site for many compounds such as nutrients or toxic compounds [41]. The digestive tract of bees is divided into three compartments, the foregut or stomodaeum, the midgut or mesenteron, and the hindgut or proctodaeum [42]. Due to the importance of food digestion and the absorption of nutrients [43], the midgut can be used as a key organ for cell biomarkers evaluation in ecotoxicological studies of the sublethal effects of pesticide exposure [44,45,46,47,48].

According to Lourencetti et al. [49], three stingless bee species showed greater sensitivity to pesticide exposure (the insecticide neonicotinoid) than the model organism Africanized *A. mellifera* used in Brazil. Thus, stingless bees should be included in toxicological evaluation programs, as Africanized honey bees do not represent the country’s vast diversity of bee species [50]. Based on this, the effects of fungicides on these native bees need to be clarified. Therefore, this study used an ultrastructural approach to evaluate the morphological alterations on a subcellular level induced by the fungicide pyraclostrobin and indications of cytotoxicity on the midgut cells after the oral exposure of *M. scutellaris* to this fungicide.

## 2. Materials and Methods

### 2.1. Meliponary

The bees used in this work were obtained from a meliponary located at the Federal University of São Carlos (UFSCar), Sorocaba campus (23°34′52.1″ S 47°31′34.7″ W), Sorocaba, Brazil. Before starting the experiments, the colonies were visually inspected to ensure a healthy status. Only colonies with similar strengths and populations that did not swarm were selected (n = 3). No chemical treatment was applied to manage the colonies, and they were kept in an urban area without pesticide application.

### 2.2. Stingless Bee Sampling

**Figure 1:**
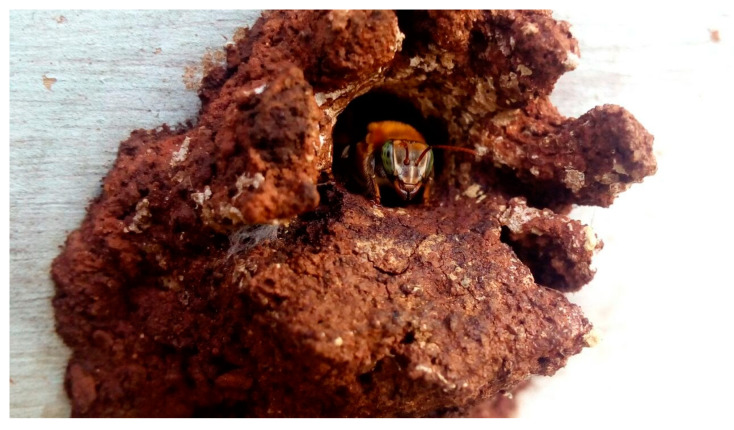
A representative picture of the study model, the *M. scutellaris* stingless bee.

Forager bees of *M. scutellaris* were sampled at the entrance of nests of three non-parental colonies from the meliponary (Section 2.1) when they returned from foraging activity (Figure 1). The collections were carried out with plastic bee cages (9 × 7 cm, 250 mL) containing 120 aeration holes (3 mm) and feeders (microtube 2 µL) filled with syrup (crystal sugar 1:1 water, *w*:*w*) on primarily sunny days at 7:30–9:00 a.m., with temperatures with a range of 15–25 °C, throughout the summer in the Southern Hemisphere in 2021. After sampling, the cages were covered with fabric to avoid stress and transferred to the “Laboratório de Ecotoxicologia e Análise de Integridade Ambiental (LEIA)” at UFSCar and placed in an incubator at a constant temperature of 28 °C (±1) and 65% (±5) relative humidity in darkness before starting the bioassay.

### 2.3. Fungicide Pyraclostrobin

The standard analytical chemical was purchased from Sigma-Aldrich (CAS Number 175013-18-0, 99.9% purity). The stock solution (1000 ng a.i./mL) was prepared using acetone as a solvent and autoclaved distilled water in proportions of 60–40%, respectively. Dilutions were performed to obtain the working solutions (0.125 ng a.i./µL and 0.005 ng a.i./µL) based on Domingues et al. [35,48]. These concentrations have been found in resources collected by bees [30,32].

### 2.4. Oral Exposure to Pyraclostrobin

For the bioassays, after bee sampling (Section 2.2), the feeders were removed from the cages two hours before starting oral exposure with two concentrations of the fungicide pyraclostrobin in syrup (0.125 ng a.i./µL—FG1; 0.005 ng a.i./µL—FG2). Then, the bees were randomly divided into fungicide-treated groups (FG1 and FG2), untreated controls (CTL), and solvent controls (CAC), with four replicates (n = 20 bees per cage); each experimental group contained 80 bees. The bees from CTL were fed syrup only, and the bees from CAC received syrup containing acetone (1% of the final volume) based on the recommendation of the OECD in 2013 [51]. Oral exposure was performed *ad libitum* over five days based on previous studies [35,48].

### 2.5. Midgut Processing for Morphological Analysis

In order to evaluate the effects of oral exposure to pyraclostrobin on *M. scutellaris*, the bees were randomly selected from all the groups (n = 6) and rendered motionless using a low temperature (4 °C) for one minute, and then dissected under a stereomicroscope at room temperature 25 °C (±1). The midguts were collected and processed for ultrastructural analysis. Light microscopy analysis was also performed according to the methodology described in the Supplementary Materials (SM). The purpose was to identify which midgut regions were more suitable for ultrastructure analysis by electron microscopy (Figure S1).

#### Transmission Electron Microscopy (TEM)

Three midguts for each experimental group were sampled, and the median region of each individual was subdivided into three portions (median subregions), fixed in a Karnovsky solution (2.5% glutaraldehyde—4% formaldehyde) in 0.1 M phosphate-buffered saline (pH 7.3) for 24 h at room temperature, and postfixed in 1% osmium tetroxide using the same buffer. Then, the midguts were washed in phosphate-buffered saline, dehydrated in a graded acetone series (50%, 75%, 90%, 95%, and 100%), and embedded in ultrapure resin (Araldite^®^). This process resulted in nine blocks per experimental group. The ultra-sections (90–60 nm) obtained from all samples were contrasted with 0.5% uranyl acetate for 20 min and lead citrate for 10 min (room temperature), and then visualized and photographed using a transmission electron microscope (Tecnai Spirit—FEI Company). Ninety regions were examined per experimental group. All steps were conducted as established in the protocol used at the Electron Microscopy Center of the Bioscience Institute (UNESP, Botucatu—Brazil), where these steps were performed.

## 3. Results

**Figure 2:**
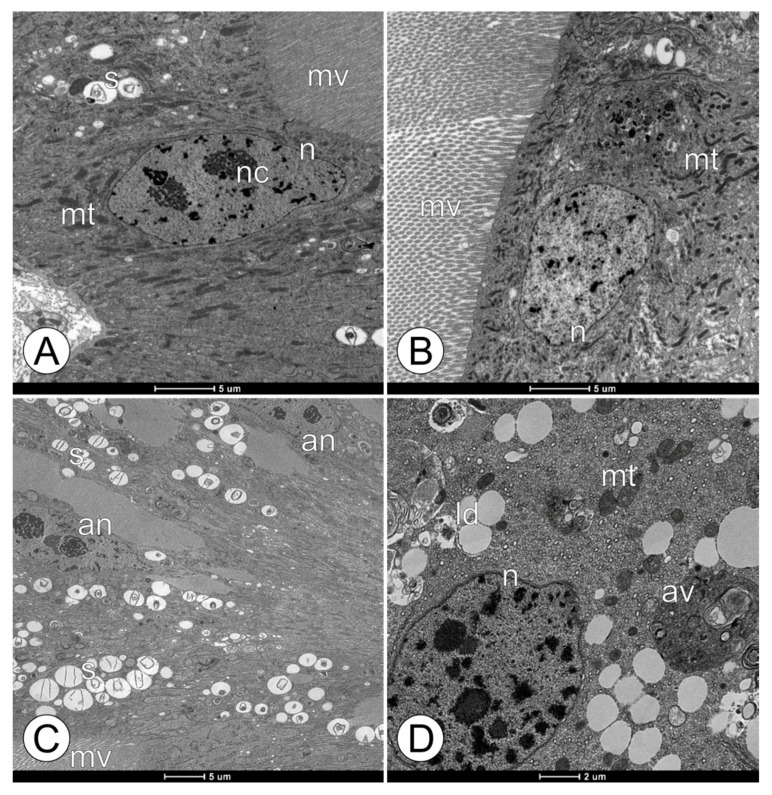
Ultrastructural midgut epithelium morphology in forager workers of *M. scutellaris* following five days of exposure to pyraclostrobin. (**A**) Untreated control—CTL; (**B**) solvent control—CAC; (**C**) pyraclostrobin (0.125 ng a.i./µL)—FG1; (**D**) pyraclostrobin (0.005 ng a.i./µL)—FG2. Altered nuclei (an), autophagic vacuole (av), lipid deposit (ld), microvilli (mv), mitochondria (mt), nuclei (n), nucleolus (nc), and spherocrystal (s).

The TEM analysis pattern performed on the midgut epitheliums of the forager workers of *M. scutellaris* from all the experimental groups is highlighted in Figure 2. Based on the analysis, the midgut epitheliums of the bees from the CTL and CAC groups were determined to be similar. The digestive cells in both exhibited well-developed microvilli containing mitochondria with a high electron density and regular morphology, as well as mitochondria in varied formats, spherocrystals, and myelin figures in average amounts for the forager’s life stage (Figure 2A,B). In contrast, the bees in the FG1 group showed large, homogeneous, electron-lucent regions in the cytoplasm of the midgut digestive cells, like cytoplasm vacuolization, while the bees in the FG2 group exhibited only small extensions of electron-lucent material in the cytoplasm (Figure 2C,D). These changes were not observed in the bees from the control groups. Additionally, digestive cells of the bees’ midguts from the FG1 group showed an altered nuclei morphology with irregular shapes and several spherocrystals, which were absent in the CTL and CAC groups (Figure 2C). The midgut digestive cells from the FG2 group exhibited autophagic vacuoles in the cytoplasm (Figure 2D).

**Figure 3:**
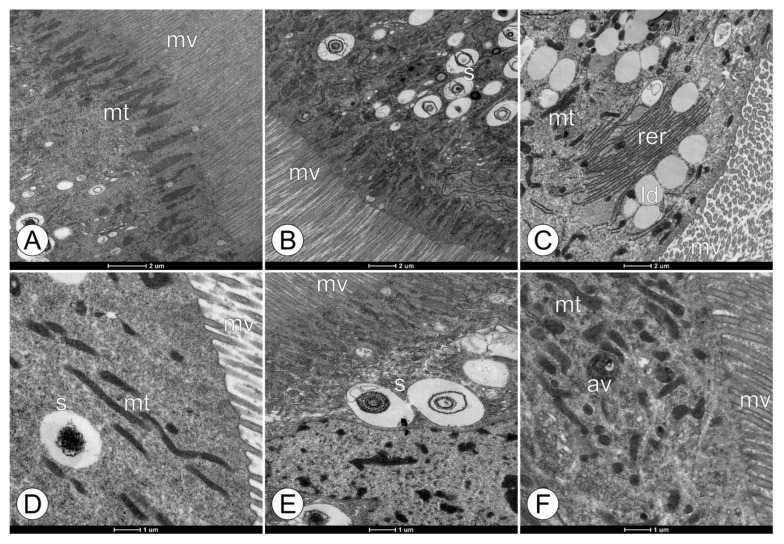
Ultrastructural changes in the apical region of midgut epithelium in forager workers of *M. scutellaris* following five days of exposure to pyraclostrobin. (**A**,**D**) Untreated control—CTL; (**B**,**E**) pyraclostrobin (0.125 ng a.i./µL)—FG1; (**C**,**F**) pyraclostrobin (0.005 ng a.i./µL)—FG2. Autophagic vacuole (av), lipid deposit (ld), microvilli (mv), mitochondria (mt), rough endoplasmic reticulum (rer), and spherocrystal (s).

Figure 3 summarizes the apical region of the digestive cells in the midgut region, highlighting the changes mentioned above (Figure 2). The midgut digestive cells in the bees from the CTL group demonstrated standard organelle morphology, well-organized microvilli, followed by a cytoplasm rich in mitochondria, and displayed some typical spherocrystals (Figure 3A,D). Similar to those found in bees from the CTL group, the bees in FG1 and FG2 revealed digestive cells with well-organized microvilli and a cytoplasm rich in mitochondria (Figure 3B,C,E,F). However, the presence of agglomerations of spherocrystals and a mischaracterized nuclei were observed when compared to the CTL group (Figure 3B,E). Similarly, the bees from the FG2 group presented autophagic vacuoles and lipid vacuoles in the cytoplasm of digestive cells, which were not seen in the CTL group (Figure 3C,F).

**Figure 4:**
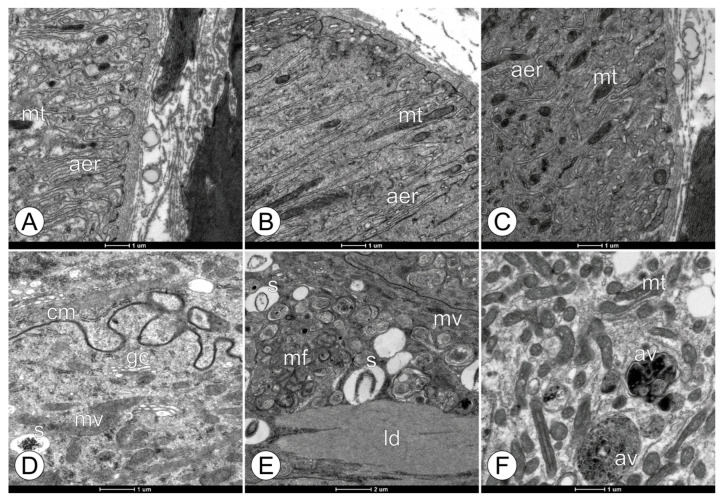
Ultrastructural changes in the basal and medial regions of midgut epithelium in forager workers of *M. scutellaris* following five days of exposure to pyraclostrobin. (**A**,**D**) Untreated control—CTL; (**B**,**E**) pyraclostrobin (0.125 ng a.i./µL)—FG1; (**C**,**F**) pyraclostrobin (0.005 ng a.i./µL)—FG2. Agranular endoplasmic reticulum (aer), autophagic vacuole (av), cytomembrane (cm), Golgi complexes (gc), lipid deposit (ld), mitochondria (mt), myelin figures (mf), and spherocrystal (s).

Regarding the basal region of the midgut digestive cells in the midgut region, the individuals from all the experimental groups did not show any morphological changes among themselves (Figure 4). The cells in this region were characterized by the presence of evident agranular endoplasmic reticulums and mitochondria associated with the membrane forming the basal labyrinth (Figure 4A–C). In the cellular medial region, the digestive cells of the bees not exposed to the fungicide pyraclostrobin (CTL and CAC) showed a large quantity of vesiculated Golgi apparatus (Figure 4D). However, the bees from the FG1 group exhibited a more significant extension of myelin figures and spherocrystals compared to those of the CTL and FG2 groups in the medial region of the digestive cells (Figure 4E). Regarding the FG2 group, the bees demonstrated the presence of autophagic vacuoles in the digestive cells (Figure 4F).

**Figure 5:**
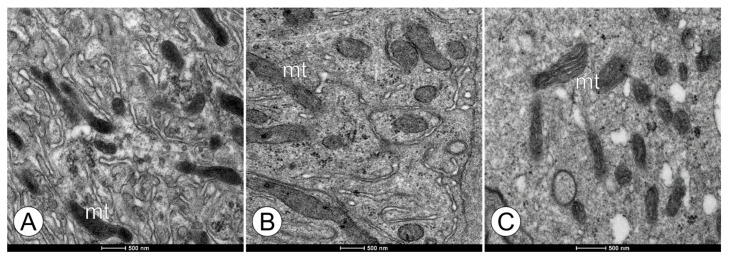
Ultrastructural morphology in the mitochondria of the digestive cells in the midgut epithelium in forager workers of *M. scutellaris* following five days of exposure to pyraclostrobin. (**A**) Untreated control—CTL; (**B**) pyraclostrobin (0.125 ng a.i./µL)—FG1; (**C**) pyraclostrobin (0.005 ng a.i./µL)—FG2. Mitochondria (mt).

**Figure 6:**
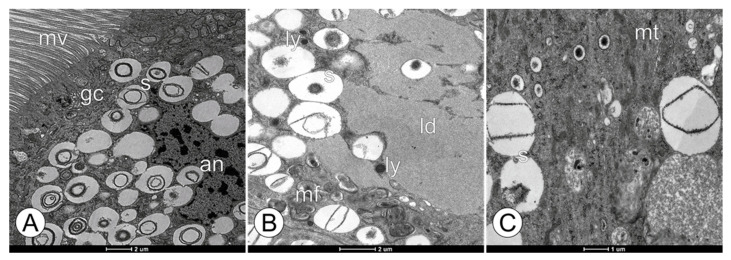
Ultrastructural changes in the spherocrystals of the digestive cells in the midgut epithelium in forager workers of *M. scutellaris* following five days of exposure to pyraclostrobin. (**A**,**B**) pyraclostrobin (0.125 ng a.i./µL)—FG1; (**C**) pyraclostrobin (0.005 ng a.i./µL)—FG2. Altered nuclei (an), Golgi complexes (gc), lipid deposit (ld), lysosomes (ly), microvilli (mv), mitochondria (mt), myelin figures (mf), and spherocrystals (s).

Based on the TEM analysis of the digestive cells, the mitochondria exhibited similar ultrastructural morphologies among the groups (Figure 5). However, the mitochondrial cristae appeared tubular and slightly dilated in the bees from the FG1 and FG2 groups, becoming more evident (Figure 5A–C). The bees in the CTL and CAC groups did not exhibit this trait and were similar. Figure 6 highlights the spherocrystals present in the FG1 and FG2 bee groups; some of them appeared unstructured, and this feature was present only in the fungicide-exposed groups.

## 4. Discussion

Pesticides have been recognized as significant stressors, contributing to honey bee colony losses [52,53]. In Brazil, the weakness and losses in colonies of Africanized *A. mellifera* are highly associated with pesticide use [54,55]. In the same way, populations of stingless bees are also at risk due to pesticide exposure [25]. According to Rondeau and Raine [56], there are significantly more knowledge gaps regarding the risk of fungicides on some bees, particularly wild bees, compared to honey bees. From this point of view, the findings of pyraclostrobin’s harmful effects on the midgut’s ultrastructure are crucial to clarify some of these gaps in native stingless bees.

The electron-lucent areas, similar to cytoplasmic vacuolization, observed in the digestive cells of *M. scutellaris* indicate a cytotoxic effect caused by exposure to the higher residual concentration of pyraclostrobin continuously ingested by these bees. Similar effects were reported in *A. mellifera* workers exposed to the fungicide iprodione (dicarboximide) at a concentration of 2 mg/kg [46] and the fungicide azoxystrobin (strobilurin) at 100 μg a.i./bee [57]. Additionally, Batista et al. [58] highlighted cellular vacuolization at the zone of differentiation above the regenerative cells in the midgut of honey bees after exposure to fungicide picoxystrobin (strobilurin) at a concentration of 0.018 ng a.i./µL.

Ultrastructural analyses revealed the presence of atypical nuclear morphology, indicating a prelude to cellular death, which was more pronounced in the bees from the FG1 group. Cytotoxic effects, such as pyknotic nuclei, were also observed in the midgut epitheliums of the adult honey bees after four days of larval exposure to the fungicide pyraclostrobin at a concentration of 4.93 ng/mL, as reported by Tadei et al. [59]. The described characteristics are associated with programmed cell death (apoptosis) [60]. These responses can act as a defense mechanism against pyraclostrobin exposure, as observed with the other stressors [44,61,62,63]. However, even with the characteristics indicative of cytotoxicity, the cells maintained intact and probably active organelles (normal morphology), such as Golgi complexes, rough endoplasmic reticulums, and mitochondria. If the oral exposure has been prolonged, the cytotoxic effects might have expanded, and these cells would likely die.

Although the mode of action of pyraclostrobin and the other strobilurin fungicides is known to involve the inhibition of the respiratory chain [33], no drastic ultra-morphological alterations were observed in the mitochondria of the bees from the FG1 and FG2 groups. These bees exhibited only slightly dilated mitochondrial cristae, suggesting that energy production could be decreased with continued exposure. According to Zick et al. [64], the cristae morphology is linked to the bioenergetic state of the mitochondria, although there are still some gaps in understanding the key factors. A study conducted by Campbell et al. [65] highlighted that honey bees increased their mitochondrial oxygen consumption rates when exposed to the strobilurin fungicide Pristine^®^ at concentrations of 5 ppm and higher. Ultrastructural alterations in the mitochondrial cristae were observed in the newly emerged workers of Africanized *A. mellifera* after exposure to thiamethoxam (0.001 ng/μL) during the larval phase [66], such as dilated mitochondria with a deformed shape and a loss of cristae. The mitochondrial cristae can vary from simple tubular structures to more complex lamellar structures merging with the inner boundary membrane, and their ultrastructural features have important implications for mitochondrial bioenergetics, biogenesis, and the role of mitochondria in apoptosis [67].

Myelin figures and autophagic vacuoles, or autophagosomes, are common in the digestive cells of the bee midgut, which exhibit a high turnover level of intracellular compounds, such as membranes and organelles, due to their multiple functions [68]. These cells synthesize digestive enzymes [69], compounds of the peritrophic matrix [70], and membrane protein transporters for nutrient absorption in the midgut [40]. The high absorption rate of digestive cells is linked to the longer striated border observed in this study, similar to what has been found in the midguts of other stingless bee species [71]

An alteration in the ultrastructure of the spherocrystal was observed. This alteration supports our hypothesis that fungicide exposure impairs cellular metabolism and causes the excretion of toxic products within the digestive cells. According to Serrão et al. [40], spherocrystals are relevant to maintaining osmoregulation, storing inorganic compounds, and preventing intoxication. According to the lesion index (severity and reversibility) adapted to bees [72], a score of one was assigned to spherocrystal alteration due to its association with the inactivation of toxic substances. Although in a scenario with continuous exposure, it could change and worsen.

In summary, our findings indicate that the fungicide pyraclostrobin clearly compromises the midgut of *M. scutellaris* at the ultrastructural level, offering another perspective on the effects of this fungicide on native stingless bees. These results reinforce our previous studies conducted by our research group [35,48,50,59,73]. Furthermore, our results further contribute to developing preventive safety measures to reduce the risks of pesticide use to bees. Regarding this issue, applying pesticides when bees are less active, considering weather conditions, establishing buffer zones, and implementing monitoring, integrated pest management, and habitat conservation measures, may reduce the risks of pesticides to bees.

## 5. Conclusions

The results of this study confirm the hypothesis that ingested pyraclostrobin induces cytotoxic effects at the ultrastructural, subcellular level of the midgut in native stingless bees. In conclusion, these cellular responses of the midgut at the tissue level may serve as a prelude to reduced bee survival rates. Therefore, it is necessary to consider the effects of fungicides on native bees and include them in protective measures to enhance regulatory decisions on risk assessment. Additionally, more studies like this would reduce the knowledge gap in how different external factors affect stingless bees.

## Data Availability

All data associated with this research publication are available from Caio Eduardo da Costa Domingues (cecdomingues@gmail.com).

## Supplementary Materials

The following supporting information can be downloaded at: https://www.mdpi.com/article/10.3390/toxics11121028/s1. Figure S1: Midgut epithelium in forager workers of *M. scutellaris*, following five days of exposure to pyraclostrobin. (A) Untreated control—CTL; (B) solvent control—CAC; (C) pyraclostrobin 0.125 ng a.i./µL—FG1; (D) pyraclostrobin 0.005 ng a.i./µL—FG2. Asterisk (a) = apocrine secretion, black arrow = cells being released into the lumen (l), muscle (m) and villi (l). N = 6 individuals per experimental group.

## References

[1] Rasmussen C., Cameron S.A. (2010). Global stingless bee phylogeny supports ancient divergence, vicariance, and long distance dispersal. Biol. J. Linn. Soc..

[2] Grüter C. (2020). Stingless Bees: Their Behaviour, Ecology and Evolution.

[3] Michener C. (2007). The Bees of the World.

[4] Eardly C.D. (2004). Taxonomic revision of the African stingless bee (Apoidea: Apidae: Apinae: Meliponini). Afr. Plant Prot..

[5] Pedro S.R.M. (2014). The stingless bee fauna in Brazil (Hymenoptera: Apidae). Sociobiology.

[6] Bueno F.G.B., Kendall L., Alves D.A., Tamara M.L., Heard T., Latty T., Gloag R. (2023). Stingless bee floral visitation in the global tropics and subtropics. Glob. Ecol. Conserv..

[7] Roubik D.W. (2006). Stingless bee nesting biology. Apidologie.

[8] Imperatriz-Fonseca V., Saraiva A.M., Gonçalves L. (2007). The Brazilian pollinators initiative and the advances for the comprehension of the role of pollinators as ecosystem services providers. Biosci. J..

[9] Imperatriz-Fonseca V.L., Alves-dos-Santos I., Santos-Filho P.S., Engels W., Ramalho M., Wilms W., Aguilar J.B.V., Pinheiro-Machado C.A., Alves D.A., Kleinert A.M.P. (2011). Checklist of bees and honey plants from São Paulo State, Brazil. Biota Neotrop..

[10] Bruckman D., Campbell D.R. (2014). Floral neighborhood influences pollinator assemblages and effective pollination in a native plant. Oecologia.

[11] Heard T.A. (1999). The role of stingless bees in crop pollination. Annu. Rev. Entomol..

[12] Slaa E.J., Sánchez Chaves L.A., Malagodi-Braga K.S., Hofstede F.E. (2006). Stingless bees in applied pollination: Practice and perspectives. Apidologie.

[13] Viana B.F., Coutinho J.G.E., Garibaldi L.A., Castagnino G.L.B., Gramacho K.P., Silva F.O. (2014). Stingless bees further improve apple pollination and production. J. Pollinat. Ecol..

[14] Giannini T.C., Alves D.A., Alves R., Cordeiro G.D., Campbell A.J., Awade M., Bento J.M.S., Saraiva A.M., Imperatriz-Fonseca V.L. (2020). Unveiling the contribution of bee pollinators to Brazilian crops with implications for bee management. Apidologie.

[15] Zulhendri F., Perera C.O., Chandrasekaran K., Ghosh A., Tandean S., Abdulah R., Herman H., Lesmana R. (2022). Propolis of stingless bees for the development of novel functional food and nutraceutical ingredients: A systematic scoping review of the experimental evidence. J. Funct. Foods..

[16] Rozman A.S., Hashim N., Maringgal B., Abdan K. (2022). A comprehensive review of stingless bee products: Phytochemical composition and beneficial properties of honey, propolis, and pollen. Appl. Sci..

[17] Choudhari M.K., Punekar S.A., Ranade R.V., Paknikar K.M. (2012). Antimicrobial activity of stingless bee (*Trigona* sp.) propolis used in the folk medicine of Western Maharashtra, India. J. Ethnopharmacol..

[18] Rao P.V., Krishnan K.T., Salleh N., Gan S.H. (2016). Biological and therapeutic effects of honey produced by honey bees and stingless bees: A comparative review. Rev. Bras. Farmacogn..

[19] Lavinas F.C., Macedo E.H.B.C., Sá G.B.L., Amaral A.C.F., Silva J.R.A., Azevedo M.M.B., Vieira B.A., Domingos T.F.S., Vermelho A.B., Carneiro C.S. (2019). Brazilian stingless bee propolis and geopropolis: Promising sources of biologically active compounds. Rev. Bras. Farmacogn..

[20] Al-Hatamleh M.A.I., Boer J.C., Wilson K.L., Plebanski M., Mohamud R., Mustafa M.Z. (2020). Antioxidant-based medicinal properties of stingless bee products: Recent progress and future directions. Biomolecules.

[21] Alves R.M.O., Carvalho C.A.L., Souza B.A., Santos W.S. (2012). Areas of natural occurrence of *Melipona scutellaris* Latreille, 1811 (Hymenoptera: Apidae) in the state of Bahia, Brazil. An. Acad. Bras. Ciênc..

[22] Medeiros V.F.L.P., Azevedo I.M., Rêgo A.C.M., Egito E.S.T., Araújo-Filho I., Medeiros A.C. (2016). Antibacterial properties and healing effects of *Melipona scutellaris* honey in MRSA-infected wounds of rats. Acta Cir. Bras..

[23] Cunha M.G., Rosalen P.L., Franchin M., Alencar S.M., Ikegaki M., Ransom T., Beutler J.A. (2016). Antiproliferative constituents of geopropolis from the bee *Melipona scutellaris*. Planta Med..

[24] ICMBio (Instituto Chico Mendes de Conservação da Biodiversidade) (2018). Livro Vermelho da Fauna Brasileira Ameaçada de Extinção.

[25] Toledo-Hernández E., Peña-Chora G., Hernández-Velázquez V.M., Lormendez C.C., Toribio-Jiménez J., Romero-Ramírez Y., León-Rodríguez R. (2022). The stingless bees (Hymenoptera: Apidae: Meliponini): A review of the current threats to their survival. Apidologie.

[26] Arena M., Sgolastra F. (2014). A meta-analysis comparing the sensitivity of bees to pesticides. Ecotoxicology.

[27] Gikas G.D., Parlakidis P., Mavropoulos T., Vryzas Z. (2022). Particularities of fungicides and factors affecting their fate and removal efficacy: A review. Sustainability.

[28] Sharma A., Kumar V., Shahzad B., Tanveer M., Sidhu G.P.S., Handa N., Kohli S.K., Yadav P., Bali A.S., Parihar R.D. (2019). Worldwide pesticide usage and its impacts on ecosystem. SN Appl. Sci..

[29] Raimets R., Bontšutšnaja A., Bartkevics V., Pugajeva I., Kaart T., Puusepp L., Pihlik P., Keres I., Viinalass H., Mänd M. (2020). Pesticide residues in beehive matrices are dependent on collection time and matrix type but independent of proportion of foraged oilseed rape and agricultural land in foraging territory. Chemosphere.

[30] Pettis J.S., Lichtenberg E.M., Andree M., Stitzinger J., Rose R., van Engelsdorp D. (2013). Crop pollination exposes honey bees to pesticides which alters their susceptibility to the gut pathogen *Nosema ceranae*. PLoS ONE.

[31] Zioga E., Kelly R., White B., Stout J.C. (2020). Plant protection product residues in plant pollen and nectar: A review of current knowledge. Environ. Res..

[32] Yoder J.A., Jajack A.J., Rosselot A.E., Smith T.J., Yerke M.C., Sammataro D. (2013). Fungicide contamination reduces beneficial fungi in bee bread based on an area-wide field study in honey bee, *Apis mellifera*, colonies. J. Toxicol. Environ. Health Part A.

[33] Bartlett D.W., Clough J.M., Godwin J.R., Hall A.A., Hamer M., Parr-Dobrzanski B. (2002). The strobilurin fungicides. Pest. Manag. Sci..

[34] Tomé H.V.V., Ramos G.S., Araujo M.F., Santana W.C., Santos G.R., Guedes R.N., Maciel C.D., Newland P.L., Oliveira E.E. (2017). Agrochemical synergism imposes higher risk to neotropical bees than to honeybees. R. Soc. Open Sci..

[35] Domingues C.E.C., Inoue L.V.B., Silva-Zacarin E.C.M., Malaspina O. (2020). Fungicide pyraclostrobin affects midgut morphophysiology and reduces survival of Brazilian native stingless bee *Melipona scutellaris*. Ecotoxicol. Environ. Saf..

[36] Prado F.S.R., Santos D.M., Almeida Oliveira T.M., Burgarelli J.A.M., Castele J.B., Vieira E.M. (2020). Determination and uptake of abamectin and difenoconazole in the stingless bee *Melipona scutellaris* Latreille (1811) via oral and topic acute exposure. Environ. Pollut..

[37] Almeida C.H.S., Haddi K., Toledo P.F.S., Rezende S.M., Santana W.C., Guedes R.N.C., Newland P.L., Oliveira E.E. (2021). Sublethal agrochemical exposures can alter honey bees’ and Neotropical stingless bees’ color preferences, respiration rates, and locomotory responses. Sci. Total Environ..

[38] Brigante J., Costa J.O., Espíndola E.L.G., Daam M.A. (2021). Acute toxicity of the insecticide abamectin and the fungicide difenoconazole (individually and in mixture) to the tropical stingless bee *Melipona scutellaris*. Ecotoxicology.

[39] Serrão J.E., Cruz-Landim C. (1996). Ultrastructure of digestive cells in stingless bees of various ages (Hymenoptera, Apidae, Meliponinae). Cytobios.

[40] Serrão J.E., Ronnau M., Neves C.A., Campos L.A., Zanuncio J.C. (2008). Ultrastructure of anterior midgut region of corbiculate bees (Hymenoptera: Apidae). Ann. Entomol. Soc. Am..

[41] Denecke S., Swevers L., Douris V., Vontas J. (2018). How do oral insecticidal compounds cross the insect midgut epithelium?. Insect Biochem. Mol. Biol..

[42] Cruz-Landim C. (2009). Abelhas: Morfologia e Função de Sistemas.

[43] Terra W.R., Ferreira C. (2020). Evolutionary trends of digestion and absorption in the major insect orders. Arthropod Struct. Dev..

[44] Motta J.V.d.O., Carneiro L.S., Martínez L.C., Bastos D.S.S., Resende M.T.C.S., Castro B.M.C., Neves M.M., Zanuncio J.C., Serrão J.E. (2023). Midgut cell damage and oxidative stress in *Partamona helleri* (Hymenoptera: Apidae) workers caused by the insecticide lambda-cyhalothrin. Antioxidants.

[45] Serra R.S., Cossolin J.F.S., Resende M.T.C.S., Castro M.A., Oliveira A.H., Martínez L.C., Serrão J.E. (2021). Spiromesifen induces histopathological and cytotoxic changes in the midgut of the honeybee *Apis mellifera* (Hymenoptera: Apidae). Chemosphere.

[46] Carneiro L.S., Martínez L.C., Gonçalves W.G., Santana L.M., Serrão J.E. (2020). Ecotoxicology and Environmental Safety the fungicide iprodione affects midgut cells of non-target honey bee *Apis mellifera* workers. Ecotoxicol. Environ. Saf..

[47] Castro M.B.A., Martinez L.C., Cossolin J.F.S., Serra R.S., Serrão J.E. (2020). Cytotoxic effects on the midgut, hypopharyngeal, glands and brain of *Apis mellifera* honey bee workers exposed to chronic concentrations of lambda-cyhalothrin. Chemosphere.

[48] Domingues C.E.C., Inoue L.V.B., Silva-Zacarin E.C.M., Malaspina O. (2020). Foragers of Africanized honeybee are more sensitive to fungicide pyraclostrobin than newly emerged bees. Environ. Pollut..

[49] Lourencetti A.P.S., Azevedo P., Miotelo L., Malaspina O., Nocelli R.C.F. (2023). Surrogate species in pesticide risk assessments: Toxicological data of three stingless bees species. Environ. Pollut..

[50] Assis J.C., Tadei R., Menezes-Oliveira V.B., Silva-Zacarin E.C.M. (2022). Are native bees in Brazil at risk from the exposure to the neonicotinoid imidacloprid?. Environ. Res..

[51] OECD (Organization for Economic Co-operation and Development) (1998). Test No. 213: Honeybees, Acute Oral Toxicity Test. OECD Guidelines for the Testing of Chemicals.

[52] Sánchez-Bayo F., Goulson D., Pennacchio F., Nazzi F., Goka K., Desneux N. (2016). Are bee diseases linked to pesticides?—A brief review. Environ. Int..

[53] Insolia L., Molinari R., Rogers S.R., Williams G.R., Chiaromonte F., Calovi M. (2022). Honey bee colony loss linked to parasites, pesticides and extreme weather across the United States. Sci. Rep..

[54] Maggi M., Antúnez K., Invernizzi C., Aldea P., Vargas M., Negri P., Brasesco C., De Jong D., Message D., Teixeira E.W. (2016). Honeybee health in South America. Apidologie.

[55] Castilhos D., Bergamo G.C., Kastelic J.P. (2021). Honey bee colony losses in Brazil in 2018-2019. Braz. J. Anim. Environ. Res..

[56] Rondeau S., Raine N.E. (2022). Fungicides and bees: A review of exposure and risk. Environ. Int..

[57] Serra R.S., Martínez L.C., Cossolin J.F.S., Resende M.T.C.S., Carneiro L.S., Fiaz M., Serrão J.E. (2023). The fungicide azoxystrobin causes histopathological and cytotoxic changes in the midgut of the honey bee *Apis mellifera* (Hymenoptera: Apidae). Ecotoxicology.

[58] Batista A.C., Domingues C.E.C., Costa M.J., Silva-Zacarin E.C.M. (2020). Is a strobilurin fungicide capable of inducing histopathological effects on the midgut and Malpighian tubules of honey bees?. J. Apic. Res..

[59] Tadei R., Menezes-Oliveira V.B., Silva-Zacarin E.C.M. (2020). Silent effect of the fungicide pyraclostrobin on the larval exposure of the non-target organism Africanized *Apis mellifera* and its interaction with the pathogen *Nosema ceranae* in adulthood. Environ. Pollut..

[60] Elmore S. (2007). Apoptosis: A review of programmed cell death. Toxicol. Pathol..

[61] Domingues C.E.C., Abdalla F.C., Balsamo P.J., Pereira B.V.R., Hausen M.A., Costa M.J., Silva-Zacarin E.C.M. (2017). Thiamethoxam and picoxystrobin reduce the survival and overload the hepato-nephrocitic system of the Africanized honeybee. Chemosphere.

[62] Carneiro L.S., Martinez L.C., Oliveira A.H., Cossolin J.F.S., Resende M.T.C.S., Gonçalves W.G., Medeiros-Santana L., Serrão J.E. (2022). Acute oral exposure to imidacloprid induces apoptosis and autophagy in the midgut of honey bee *Apis mellifera* workers. Sci. Total Environ..

[63] Gao J., Guo Y., Chen J., Diao Q.-Y., Wang Q., Dai P.-L., Zhang L., Li W.-M., Wu Y.-Y. (2023). Acute oral toxicity, apoptosis, and immune response in nurse bees (*Apis mellifera*) induced by flupyradifurone. Front. Physiol..

[64] Zick M., Rabl R., Reichert A.S. (2009). Cristae formation—Linking ultrastructure and function of mitochondria. Biochim. Biophys. Acta, Mol. Cell Res..

[65] Campbell J.B., Nath R., Gadau J., Fox T., Degrandi-Hoffman G., Harrison J.F. (2016). The fungicide Pristine^®^ inhibits mitochondrial function in vitro but not flight metabolic rates in honey bees. J. Insect Physiol..

[66] Friol P.S., Catae A.F., Tavares D.A., Malaspina O., Roat T.C. (2017). Can the exposure of *Apis mellifera* (Hymenoptera, Apiadae) larvae to a field concentration of thiamethoxam affect newly emerged bees?. Chemosphere.

[67] Frey T.G., Mannella C.A. (2000). The internal structure of mitochondria. Trends Biochem. Sci..

[68] Oliveira A.H., Gonçalves W.G., Fernandes K.M., Barcellos M.S., Sampaio W.M.S., Lopes M.P., Martins G.F., Serrão J.E. (2019). Morphology and morphometry of the midgut in the stingless bee *Friesella schrottkyi* (Hymenoptera: Apidae). Insects.

[69] Jimenez D.R., Gilliam M. (1989). Age-related changes in midgut ultrastructure and trypsin activity in the honey bee, *Apis mellifera*. Apidologie.

[70] Teixeira A.D., Marques-Araujo S., Zanuncio J.C., Serrão J.E. (2015). Peritrophic membrane origin in adult bees (Hymenoptera): Immunolocalization. Micron.

[71] Serrão J.E., Cruz-Landim C. The striated border of digestive cells in adult stingless bees (Hymenoptera, Apidae, Meliponinae) Cytobios 1995, 83, 229–235. 83.

[72] Grella T.C., Soares-Lima H.M., Malaspina O., Nocelli R.C.F. (2019). Semi-quantitative analysis of morphological changes in bee tissues: A toxicological approach. Chemosphere.

[73] Inoue L.V.B., Domingues C.E.C., Gregorc A., Silva-Zacarin E.C.M., Malaspina O. (2022). Harmful effects of pyraclostrobin on the fat body and pericardial cells of foragers of Africanized honey bee. Toxics.

