# Supplementary material for: Ultrastructural Changes in the Midgut of Brazilian Native Stingless Bee Melipona scutellaris Exposed to Fungicide Pyraclostrobin
Source: Toxics. 2023 Dec 18;11(12):1028. doi: 10.3390/toxics11121028 (PMC10748086; doi:10.3390/toxics11121028)
Supplement: Supplementary file 1 [file toxics-11-01028-s001.zip › toxics-2758400-supplementary.pdf]

toxics

Supplementary Materials: Ultrastructural Changes in the Midgut of Brazilian Native Stingless Bee *Melipona scutellaris* Exposed to Fungicide Pyraclostrobin

Caio E. C. Domingues, Lais V. B. Inoue, Aleš Gregorc, Leticia S. Ansaloni, Osmar Malaspina and Elaine C. Mathias da Silva

The sampled midguts were fixed in 4% paraformaldehyde with phosphate-buffered saline (PBS) at a concentration of 0.1 mol L^{1} and pH 7.4 and kept at 4 °C for 24 hours. Subsequently, they underwent dehydration with ethanol following the method established in the laboratory's protocol. After that, the organs were embedded in historesin (Leica Historesin Embedding Kit) according to the manufacturer's instructions and cut into 6 mm thick histological sections using a microtome (LEICA RM2255). Lastly, the slides containing the histological sections were stained with hematoxylin and eosin, sealed with a mounting medium, and analyzed under a microscope.

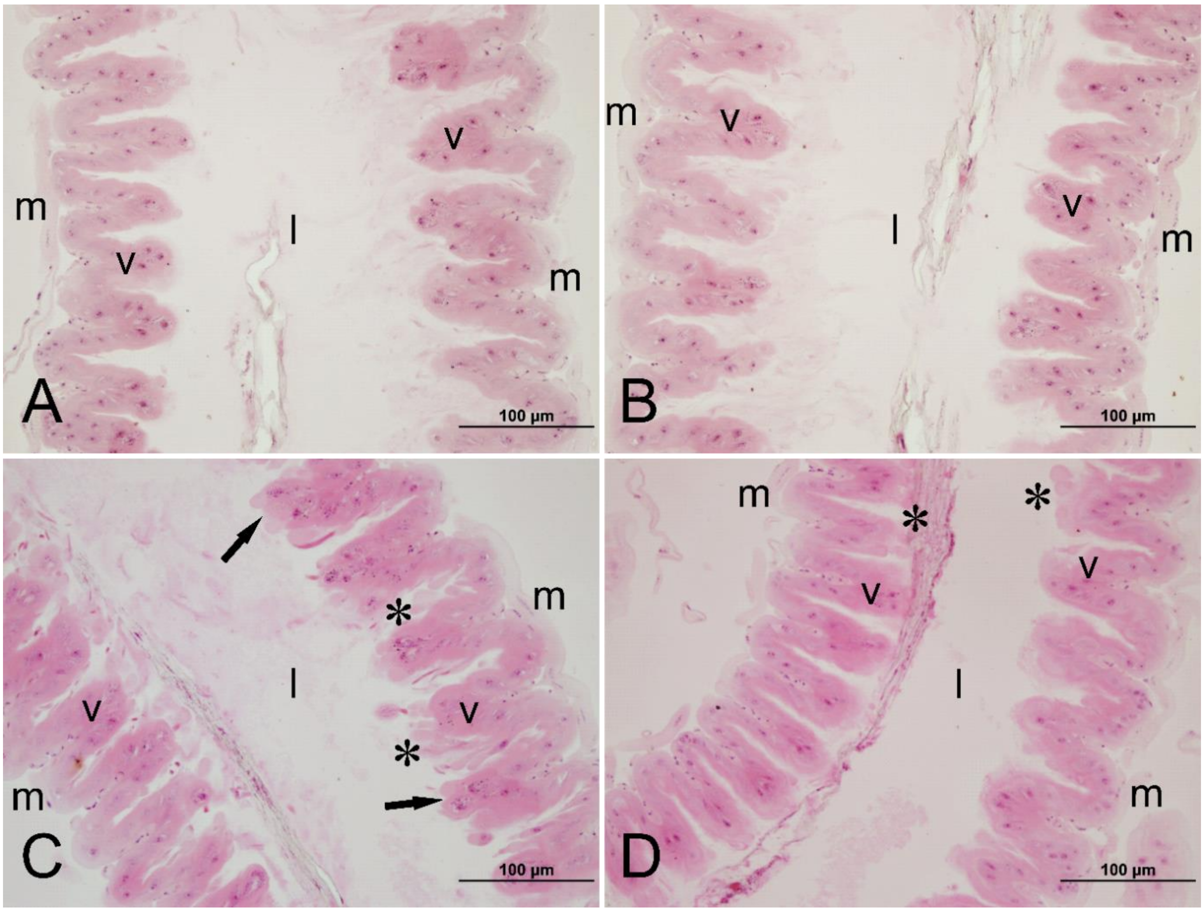

Figure S1. Midgut epithelium in forager workers of *M. scutellaris*, following five days of exposure to pyraclostrobin. (**A**) Untreated control - CTL; (**B**) solvent control - CAC; (**C**) pyraclostrobin 0.125 ng a.i./µL - FG1; (**D**) pyraclostrobin 0.005 ng a.i./µL - FG2. Asterisk (a) = apocrine secretion, black arrow = cells being released into the lumen (l), muscle (m) and villi (l). N = 6 individuals per experimental group.
